# Relationship of circulating insulin-like growth factor-I and binding proteins 1–7 with mammographic density among women undergoing image-guided diagnostic breast biopsy

**DOI:** 10.1186/s13058-019-1162-8

**Published:** 2019-07-23

**Authors:** Manila Hada, Hannah Oh, Ruth M. Pfeiffer, Roni T. Falk, Shaoqi Fan, Maeve Mullooly, Michael Pollak, Berta Geller, Pamela M. Vacek, Donald Weaver, John Shepherd, Jeff Wang, Bo Fan, Amir Pasha Mahmoudzadeh, Serghei Malkov, Sally Herschorn, Louise A. Brinton, Mark E. Sherman, Gretchen L. Gierach

**Affiliations:** 1 National Cancer Institute, National Institutes of Health, Bethesda, MD USA; 2 Division of Health Policy and Management, College of Health Sciences, Korea University, Seoul, Republic of Korea; 3 Royal College of Surgeons in Ireland, Dublin, Ireland; 4 McGill University, Montreal, QC Canada; 5 University of Vermont and Vermont Cancer Center, Burlington, VT USA; 6 University of Hawaii, Honolulu, Hawaii USA; 7 Graduate School of Medicine, Hokkaido University, Sapporo, Japan; 8 University of California San Francisco, San Francisco, CA USA; 9 Mayo Clinic, Jacksonville, FL USA

**Keywords:** Mammographic density, Breast density, Insulin-like growth factor, Insulin-like growth factor binding proteins, Breast neoplasm

## Abstract

**Background:**

Mammographic density (MD) is a strong breast cancer risk factor that reflects fibroglandular and adipose tissue composition, but its biologic underpinnings are poorly understood. Insulin-like growth factor binding proteins (IGFBPs) are markers that may be associated with MD given their hypothesized role in breast carcinogenesis. IGFBPs sequester IGF-I, limiting its bioavailability. Prior studies have found positive associations between circulating IGF-I and the IGF-I:IGFBP-3 ratio and breast cancer risk. We evaluated the associations of IGF-I, IGFBP-3, and six other IGFBPs with MD.

**Methods:**

Serum IGF measures were quantified in 296 women, ages 40–65, undergoing diagnostic image-guided breast biopsy. Volumetric density measures (MD-V) were assessed in pre-biopsy digital mammograms using single X-ray absorptiometry. Area density measures (MD-A) were estimated by computer-assisted thresholding software. Age, body mass index (BMI), and BMI^2^-adjusted linear regression models were used to examine associations of serum IGF measures with MD. Effect modification by BMI was also assessed.

**Results:**

IGF-I and IGFBP-3 were not strongly associated with MD after BMI adjustment. In multivariable analyses among premenopausal women, IGFBP-2 was positively associated with both percent MD-V (*β* = 1.49, *p* value = 0.02) and MD-A (*β* = 1.55, *p* value = 0.05). Among postmenopausal women, positive relationships between IGFBP-2 and percent MD-V (*β* = 2.04, *p* = 0.003) were observed; the positive associations between IGFBP-2 and percent MD-V were stronger among lean women (BMI < 25 kg/m^2^) (*β* = 5.32, *p* = 0.0002; *p* interaction = 0.0003).

**Conclusions:**

In this comprehensive study of IGFBPs and MD, we observed a novel positive association between IGFBP-2 and MD, particularly among women with lower BMI. In concert with in vitro studies suggesting a dual role of IGFBP-2 on breast tissue, promoting cell proliferation as well as inhibiting tumorigenesis, our findings suggest that further studies assessing the role of IGFBP-2 in breast tissue composition, in addition to IGF-1 and IGFBP-3, are warranted.

**Electronic supplementary material:**

The online version of this article (10.1186/s13058-019-1162-8) contains supplementary material, which is available to authorized users.

## Background

Increased mammographic breast density (MD) is an established risk factor for breast cancer [1, 2], which reflects the relative proportions of fibroglandular and adipose tissue content within the breast [3, 4]. However, most women with high MD neither have prevalent tumor nor will develop one in future. The determinants of MD and the mechanisms by which it increases the risk of breast cancer are poorly understood. Identifying factors associated with MD could elucidate the mechanisms by which breast density contributes to the risk of breast cancer and may improve risk prediction. Several lines of evidence have demonstrated the role of the insulin-like growth factor (IGF) system in breast cancer as either a tumor suppressor or promoter [5–7]. IGF proteins may be an underlying link between MD and the risk of breast cancer.

**Fig. 1:**
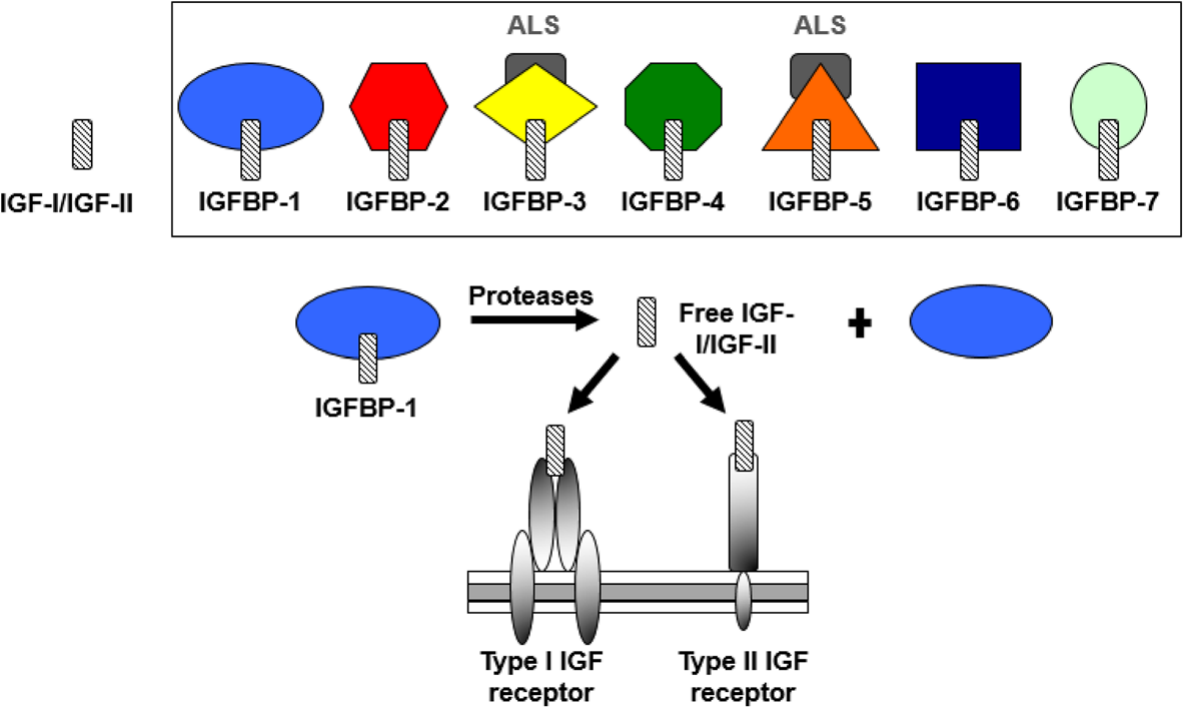
Schematic representation of the insulin-like growth factor (IGF) system. The IGF system consists of IGF-I and IGF-II, IGF binding proteins (IGFBP-1 to7), and type I and II IGF receptors. IGFBPs have high affinity to IGFs and modulate their access to IGF receptors. IGF proteases dissociate the IGFBP-IGF complex, and the freed IGFs can then bind to the IGF receptors, activating downstream signaling pathways: the phosphatidylinositol-3-kinase and RAS-extracellular signal-regulated kinase pathways. ALS: Acid Labile Subunit

The insulin-like growth factor (IGF) system comprises transmembrane growth factor receptors, growth factor ligands (IGFs), IGF binding proteins (IGFBPs), proteases, and IGFBP-related proteins (IGFBP-rP) [8–10] (Fig. 1). IGF-I is produced by the liver in response to growth hormone produced by the pituitary gland. Bioavailability of IGF-I is regulated by numerous IGFBPs. IGFBPs have high affinity to IGFs and modulate their access to IGF receptors. IGF proteases dissociate the IGFBP-IGF complex and the freed IGFs can then bind to the IGF receptors, activating downstream signaling pathways: the phosphatidylinositol-3-kinase and RAS-extracellular signal-regulated kinase pathways [11]. Activation of these tyrosine kinase pathways plays a significant role in cell proliferation, differentiation, and metabolism. Indeed, dysregulated IGF-axis proteins have been shown to play a critical role in carcinogenesis of several human cancers including breast cancer [12].

Laboratory studies suggest that IGF-I may promote breast tumorigenesis by stimulating cell proliferation and inhibiting apoptosis [10, 13]. A pooled analysis of 17 prospective epidemiologic studies showed that circulating IGF-I was associated with a 25% increased risk of breast cancer comparing women from the highest quintile to the lowest quintile, and this association did not vary by menopausal status [14]. Higher circulating concentrations of IGF-I as well as the ratio of IGF-I:IGFBP-3 have been shown to be associated with higher MD in premenopausal women in some studies [15–18], whereas others have not observed an association [16, 19–22]. Among postmenopausal women, there is little evidence for a relationship between IGF-I, IGF-I:IGFBP-3, and MD [16–19, 21–24].

While prior studies have largely focused on the role of IGF-I, IGFBP-3, and the ratio of IGF-I:IGFBP-3 in MD, the relationship of other IGFBPs (IGFBP-1, 2, 4, 5, 6, and 7 and their ratio to IGF-I) with MD has not been elucidated. Epidemiological studies have not extensively explored the associations of other IGFBPs (IGFBP 4, 5, 6, and 7) and breast cancer risk, with the exception of IGFBP-2, where findings have been inconsistent [25–27]. Laboratory and clinical data also suggest a role for IGFBPs in mammary gland development and breast carcinogenesis [28–34]. Although IGFBPs have significant sequence homology, they have different structures, binding patterns, and affinities [35]. IGFBPs are also known to exert IGF-dependent and independent actions [36, 37], with distinct functions in the breast [38]. Thus, in order to fully understand the role of the IGF system in MD, a panel of IGBPs may need to be investigated. We therefore assessed the relationship between serum levels of IGF-I and seven of its binding proteins with area and volume measures of MD in a cross-sectional study of women referred for image-guided diagnostic breast biopsy.

## Methods

### Study population

The National Cancer Institute (NCI) Breast Radiology Evaluation and Study of Tissues (BREAST) Stamp Project is a cross-sectional study of MD conducted among 465 women, aged 40 to 65 years, who were referred for diagnostic image-guided breast biopsy based on an abnormal mammogram from 2007 through 2010 at the University of Vermont (UVM) College of Medicine and University of Vermont Medical Center as described previously [39]. Participants had no prior history of breast cancer or receiving cancer treatment, had not undergone breast surgery within 1 year, did not have breast implants, and were not taking breast cancer chemoprevention [39]. A standard self-administered questionnaire collected information on participants’ health history.

Out of 465 women who consented to participate in the study, at least 1 vial of serum was collected from 346 (74%) women. Of these 346 women, 21 with missing mammographic density data and 29 who were current hormone users (menopausal hormone therapy/oral contraceptives) were excluded from the study. This resulted in a population of 296 women (193 premenopausal and 103 postmenopausal) eligible for serum IGF and IGFBP protein measurement.

### Mammographic density assessment

Digital raw mammogram images were sent to the University of California at San Francisco (UCSF) for quantitative volumetric and area density assessment [39]. This analysis was restricted to prebiopsy craniocaudal views of the ipsilateral breast. The mammogram closest in time prior to breast biopsy date was selected for this analysis. Area measures of density (MD-A) were estimated by an experienced trained reader with demonstrated reliability [40, 41] using UCSF’s computer-assisted thresholding software which is comparable to other validated methods [40, 42]. Absolute dense area (cm^2^) was measured by setting a pixel threshold for dense tissue. Percentage MD was calculated by dividing absolute dense breast area by total breast area and multiplying by 100. Single X-ray absorptiometry (SXA), in which a density phantom was affixed to the mammographic compression paddle and included in the X-ray field, was used to estimate breast density as an absolute and percent fibroglandular volume (MD-V) (cm^3^) as described previously [43]. Previous reproducibility of SXA MD measures demonstrated a repeatability SD of 2%, with a 2% accuracy for the entire thickness and density ranges [43]. The mean difference in time between age at biopsy and age at mammogram was 0.048 year; thus, on average, there was less than 6 months between age at biopsy and mammogram.

### Blood collection and laboratory assay

Whole blood samples were collected using standard techniques, allowed to clot for 30 min, and processed at the UVM General Clinical Research Center. Samples were centrifuged at 3000 rpm for 15 min, and serum was aliquoted into 2.0-mL cryovials and frozen at − 80 °C until shipment to the NCI biorepository, where vials are stored in liquid nitrogen. Serum concentrations (ng/mL) of IGF-I, IGFBP-1, IGFBP-2 and IGFBP-3 were measured as previously described [44–46]. IGF-I and IGFBP-3 were measured using chemiluminescence immunoassay (CLIA) from Immunodiagnostic Systems Ltd. (Boldon, UK). IGFBP-1, 2, 4, 5, 6, and 7 were measured by enzyme-linked immunosorbent assay using reagents from ALPCO (Salem, NH), Ansh Labs LLC (Webster, TX), Raybiotech Inc. (Norcross, GA), and R&D Systems Inc. (Minneapolis, MN). Measurement of these proteins required 400 μl of serum from each participant for duplicate measurements for each assay. The average of the duplicate measurements was used as the summary measure in the analysis. To monitor the assay reliability, 32 masked quality control samples (10% of total samples) were included within and across batches. Within batch and between batches’ coefficients of variation (CV) for IGF-I and IGFBP-1–7 were all < 8% and intraclass correlations (ICC) for all were above 92% (Table 1).

**Table 1:** Distribution of circulating IGF-I and IGFBPs 1–7 (ng/ml) among BREAST Stamp Project participants, stratified by menopausal status

IGF measure	QC measures		Premenopausal (*N* = 193)			Postmenopausal (*N* = 103)		
	CV (%)	ICC (%)	Median^1^	10th percentile	90th percentile	Median	10th percentile	90th percentile
IGF-I	2.03	99.6	120	83.7	182	111	72.5	143
IGFBP-1^2^	7.50	99.0	2.05	0.03	6.39	1.86	0.03	7.31
IGFBP-2	3.75	99.4	341	177	639	357	149	616
IGFBP-3	6.19	92.2	3502	2715	4455	3519	2676	4508
IGFBP-4	5.70	95.3	130	99	176	156	117	218
IGFBP-5	5.32	96.1	387	280	508	402	291	521
IGFBP-6	5.45	94.2	188	143	240	176	128	238
IGFBP-7	5.43	92.2	105	86.2	134	115	99.1	139

*CV* coefficient of variation, *ICC* intraclass correlation coefficient, *IGF* insulin-like growth factor, *IGFBP* IGF binding protein

^1^Median and 10 and 90th percentiles were calculated using untransformed IGF measures

^2^For IGFBP-1, *N* = 91 samples were below the lower limit of detection (LLOD) and were reassigned as ½ LLOD (0.025)

### Statistical analysis

Analyses were conducted for women overall and stratified by menopausal status as the level of several IGF and MD measures differed by menopausal status. Median and 10th and 90th percentiles of analyte concentrations were calculated using untransformed IGF measures. For IGFBP-1, 91 samples were below the lower limit of detection (LLOD) and were reassigned as ½ of LLOD (0.025 ng/ml).

To study the relationship between IGF measures and participant characteristics, IGF measures were logarithmically transformed to normalize the distributions, and geometric mean IGF concentrations and 95% confidence intervals were estimated in the analysis of variance (ANOVA) models. The characteristics examined were age at biopsy (premenopausal < 45, 45–49, ≥ 50 years; postmenopausal <54, 55–59, ≥ 60 years), education (≤ high school graduate or GED; some college/technical school; college or post-college graduate), body mass index (BMI) (< 25, 25–29.9, ≥ 30 kg/m^2^), age at menarche (≤ 12, 13, ≥ 14 years), age at first birth (nulliparous/30+ years, < 30 years), oral contraceptive use (never, former), menopausal hormone therapy use (never, former), family history of breast cancer in a first-degree relative (none, 1, or more), age at menopause (< 45, 45–49, ≥ 50 years), cigarette smoking (never, former, current), breast biopsy prior to enrollment (never, ever), and biopsy diagnosis (benign non-proliferative, proliferative with/without atypia, in situ/invasive). Trends across categories of reproductive and other risk factors were calculated by treating the categories as ordinal variables. Heterogeneity between categories was assessed by *F* tests arising from the ANOVA. The participant characteristics that were evaluated as confounders were associated with both the IGF and MD measures. Age, BMI, and cigarette smoking were previously identified as being associated with MD [39], and as they were also associated with IGF measures, we considered these factors as potential confounders.

Initially, age and categorical BMI-adjusted linear regression models were used to examine associations of serum IGF measures with measures of MD-A, MD-V, non-dense volume, and non-dense area as the dependent variables (Additional file 1: Tables S4, S5, and S7). To better capture potential confounding by BMI, we added a quadratic term for BMI, resulting in a final multivariate linear regression model adjusting for age, BMI, and BMI^2^; for these analyses, 4 pre- and 2 postmenopausal women with BMI > 45 kg/m^2^ were excluded, resulting in a final analytic population of 189 pre- and 101 postmenopausal women. MD measures were square-root transformed to normalize the distributions. Adjustment for cigarette smoking did not substantially change the estimates, and, as such, smoking was not included in the final models. We examined individual IGF measures, the ratio of IGF-I and individual IGFBPs, and the ratio of IGF-I and total IGFBPs (sum of molar concentration of individual IGFBPs). In sensitivity analyses, we examined the relation between serum IGF measures and MD after excluding women whose biopsy diagnosis included in situ or invasive breast carcinoma (*N* = 51). Effect modification by BMI was also assessed.

Factor analysis was used to evaluate the covariation among IGF measures in pre- and postmenopausal women (PROC FACTOR, SAS Institute INC., Cary NC). Factor analysis allows one to represent covariance relationships contained in several, correlated variables (e.g., IGF measures) in terms of a few (unobserved) factors [47]. To obtain the factors, we repartitioned the shared variance and the unique variance for each observed variable into linear combinations (principal components) and used Kaiser’s rule [48] to decide how many factors to retain in the model. For ease of interpretation, we derived uncorrelated factors through rotation. Factor scores were calculated for each woman and used in quintiles in linear regression to estimate the association between the factor and MD measures. We estimated factors using data from all women; however, basic patterns did not change when factors were computed separately for pre- and postmenopausal women.

All statistical tests were two-sided with probability values of < 0.05 considered to be statistically significant. All the analyses were conducted with SAS software, version 9.4 (SAS Institute INC., Cary, NC).

## Results

### Participant characteristics

Overall, 94% of subjects were white and 65% (*N* = 193) of the study participants were premenopausal (Additional file 1: Table S1). Among premenopausal women, 50.3% had BMI < 25 kg/m^2^, and among postmenopausal women, 39.8% had BMI < 25 kg/m^2^. The majority of pre- (51%) and postmenopausal (64.1%) women had age at first birth < 30 years, and 23.3% of pre- and 21.4% of postmenopausal women were nulliparous. Most pre- (88.5%) and postmenopausal (65%) women had never used menopausal hormone therapy (Additional file 1: Table S1).

### Distribution of IGF and IGFBPs in women stratified by menopausal status

The correlations between the IGF measures are presented in Additional file 1: Table S2. Distributions of the IGF measures are reported in Table 1. In both pre- and postmenopausal women, IGFBP-1 had the lowest serum concentration and IGFBP-3 had the highest serum concentration (Table 1). Median circulating levels of IGF-I were significantly higher in premenopausal (120 ng/ml) compared with postmenopausal women (111 ng/ml); *p* value < 0.0001 (Table 1). Circulating concentrations of IGFBP-4 and IGFBP-7 were significantly higher among postmenopausal (*p* values < 0.0001 for both), and IGFBP-6 was higher among premenopausal women (*p* value = 0.02). No statistically significant differences by menopausal status were observed for IGFBP-1, IGFBP-2, IGFBP-3, or IGFBP-5 levels. In analyses restricted to participants whose biopsies yielded benign biopsy diagnoses (86.6% and 75.7% of pre- and postmenopausal participants, respectively), similar patterns of IGF concentrations by menopausal status were observed (Additional file 1: Table S3).

### Relationship of circulating IGF measures with select participant characteristics

Geometric mean concentrations of IGF measures by select participant characteristics are shown in Tables 2 and 3 for pre- and postmenopausal women, respectively.

**Table 2:** Relationship of circulating unadjusted geometric mean concentrations of IGF-I and IGFP-1–7 (ng/mL) with select participant characteristics in premenopausal women

Characteristics	*N*	IGF-I	IGFBP-1	IGFBP-2	IGFBP-3	IGFBP-4	IGFBP-5	IGFBP-6	IGFBP-7
		Mean^1^ (95% CI)	Mean (95% CI)	Mean (95% CI)	Mean (95% CI)	Mean (95% CI)	Mean (95% CI)	Mean (95% CI)	Mean (95% CI)
Age at biopsy (years)									
< 45	56	131 (121–142)	1.08 (0.46–1.71)	340 (298–381)	3499 (3321–3678)	135 (126–143)	402 (378–425)	178 (167–189)	105 (100–110)
45–49	83	123 (114–131)	0.78 (0.40–1.17)	344 (311–378)	3393 (3243–3542)	130 (123–137)	390 (366–414)	185 (178–193)	107 (102–111)
≥ 50	54	112 (104–120)	0.60 (0.21–0.99)	308 (263–354)	3511 (3328–3694)	128 (121–135)	363 (338–388)	199 (187–210)	111 (105–117)
*p* trend (*p* het)		*0.005 (0.02)*	0.18 (0.88)	0.30 (0.4)	0.94 (0.53)	0.25 (0.50)	*0.04* (0.11)	*0.009 (0.03)*	0.15 (0.31)
Education									
≤ High school grad/GED	27	107 (94–121)	3.69 (2.46-4.92)	326 (264–388)	3509 (3198–3820)	132 (120–144)	336 (307–365)	191 (178–205)	106 (96–115)
Some college/tech school	37	122 (109–135)	2.42 (1.66-3.18)	277 (231–323)	3483 (3245–3721)	129 (120–138)	376 (351–401)	191 (181–201)	101 (95–107)
≥ College graduate	129	125 (119–131)	3.42 (2.95-3.88)	352 (324–380)	3438 (3326–3549)	131 (126–137)	400 (381–419)	185 (177–192)	110 (106–113)
*p* trend (*p* het)		*0.02* (0.05)	0.77 (0.11)	0.11 *(0.03)*	0.58 (0.86)	0.96 (0.89)	*0.001 (0.005)*	0.37 (0.62)	0.12 (0.05)
Body mass index (kg/m2)									
< 25	97	124 (117–131)	2.06 (1.34–2.79)	399 (364–433)	3343 (3200–3485)	128 (121–134)	396 (375–418)	185 (177–192)	107 (103–112)
25.0–29.9	48	122 (111–133)	0.91 (0.35–1.48)	340 (297–383)	3483 (3292–3675)	133 (125–141)	380 (357–404)	189 (177–201)	107 (102–113)
≥ 30	48	118 (107–128)	0.10 (0.04–0.16)	225 (200–250)	3670 (3506–3833)	136 (127–145)	370 (342–398)	189 (176–201)	108 (102–113)
*p* trend (*p* het)		0.31 (0.59)	*< 0.0001 (< 0.0001)*	*< 0.0001 (< 0.0001)*	*0.007 (0.03)*	0.11 (0.27)	0.12 (0.29)	0.53 (0.76)	0.98 (0.99)
Age at menarche (years)									
≤ 12	69	122 (112–131)	0.79 (0.38–1.21)	320 (283–358)	3561 (3394–3728)	136 (129–144)	381 (351–411)	187 (177–197)	113 (107–119)
13	73	122 (114–130)	0.99 (0.47–1.51)	343 (304–382)	3449 (3287–3611)	129 (123–136)	387 (367–401)	184 (175–193)	106 (102–110)
≥ 14	48	123 (114–132)	0.58 (0.19–0.97)	335 (290–380)	3342 (3162–3522)	128 (119–136)	394 (373–415)	191 (179–203)	104 (98–110)
*p* trend (*p* het)		0.83 (0.97)	0.55 (0.71)	0.58 (0.67)	0.09 (0.23)	0.12 (0.25)	0.49 (0.78)	0.71 (0.65)	*0.02* (0.05)
Age at first birth (years)									
Nulliparous/ ≥ 30 years	94	118 (111–126)	0.83 (0.44–1.22)	338 (302–373)	3324 (3188–3461)	132 (125–139)	392 (370–414)	184 (176–192)	108 (104–113)
< 30 years	98	126 (119–133)	0.77 (0.42–1.12)	329 (299–359)	3593 (3459–3727)	130 (124–135)	379 (361–398)	191 (183–199)	107 (103–111)
*p* het		0.14	0.81	0.70	*0.007*	0.60	0.39	0.22	0.61
Parity									
Nulliparous (0)	45	111 (100–122)	0.55 (0.15–0.96)	329 (274–383)	3251 (3052–3450)	130 (121–139)	378 (350–407)	179 (168–191)	102 (97–106)
1	20	122 (108–135)	0.51 (0.003–1.02)	272 (220–325)	3414 (3124–3704)	136 (119–154)	394 (328–460)	180 (165–197)	116 (101–132)
2	82	127 (119–134)	0.81 (0.41–1.21)	344 (309–378)	3528 (3388–3667)	128 (121–135)	390 (370–409)	190 (182–199)	109 (105–114)
≥ 3	46	126 (115–137)	1.36 (0.54–2.18)	346 (300–391)	3557 (3340–3774)	135 (127–142)	382 (354–411)	191 (179–204)	106 (101–111)
*p* trend (*p* het)		*0.02* (0.08)	0.05 (0.39)	0.35 (0.25)	*0.02* (0.10)	0.76 (0.53)	0.81 (0.90)	0.09 (0.35)	0.35 (0.05)
Characteristics	*N*	IGF-I	IGFBP-1	IGFBP-2	IGFBP-3	IGFBP-4	IGFBP-5	IGFBP-6	IGFBP-7
		Mean (95% CI)	Mean (95% CI)	Mean (95% CI)	Mean (95% CI)	Mean (95% CI)	Mean (95% CI)	Mean (95% CI)	Mean (95% CI)
Oral contraceptive use									
Never	28	124 (108–139)	0.71 (0.06–1.35)	339 (280–399)	3372 (3108–3637)	123 (110–135)	397 (354–439)	196 (182–210)	104 (97–111)
Former	165	122 (116–127)	0.82 (0.53–1.10)	331 (307–356)	3471 (3366–3575)	132 (128–137)	384 (369–399)	185 (179–192)	108 (105–111)
*p* het		0.80	0.76	0.81	0.48	0.12	0.54	0.19	0.31
Menopausal hormone use									
Never	169	123 (118–128)	0.88 (0.58–1.18)	342 (317–366)	3448 (3349–3546)	130 (126–135)	387 (371–402)	189 (183–195)	107 (104–111)
Former	22	117 (100–133)	0.41 (−0.02–0.84)	267 (210–324)	3555 (3153–3958)	132 (118–146)	372 (338–407)	177 (157–196)	109 (101–118)
*p* het		0.45	0.15	*0.03*	0.50	0.81	0.53	0.18	0.69
Family history of breast cancer in a first-degree relative									
None	145	124 (118–130)	0.74 (0.46–1.03)	331 (305–357)	3460 (3343–3577)	131 (126–136)	390 (373–407)	186 (179–192)	107 (103–111)
1 or more	47	115 (108–123)	1.08 (0.45–1.71)	335 (286–385)	3426 (3258–3594)	131 (124–138)	376 (351–400)	189 (179–202)	109 (104–114)
*p* het		0.15	0.33	0.88	0.77	0.93	0.39	0.52	0.64
Cigarette smoking									
Never	100	126 (119–134)	0.89 (0.49–1.30)	339 (306–372)	3462 (3323–3601)	135 (129–141)	402 (383–421)	189 (181–198)	109 (105–113)
Former	65	116 (109–124)	0.77 (0.36–1.18)	322 (283–360)	3417 (3264–3570)	123 (115–131)	368 (343–393)	182 (172–192)	105 (99–111)
Current	20	118 (101–136)	0.54 (−0.08–1.16)	357 (292–423)	3566 (3215–3917)	141 (129–153)	346 (313–379)	195 (182–207)	108 (98–118)
*p* trend (*p* het)		0.12 (0.19)	0.38 (0.11)	0.98 (0.65)	0.79 (0.70)	0.58 (*0.01*)	*0.005 (0.02)*	0.85 (0.36)	0.45 (0.42)
Breast biopsy prior to enrollment									
Never	130	122 (116–128)	0.71 (0.43–0.99)	321 (295–347)	3433 (3311–3556)	132 (126–137)	388 (371–405)	184 (177–191)	109 (105–113)
Ever	63	122 (113–131)	1.02 (0.45–1.58)	357 (311–402)	3504 (3348–3660)	129 (123–136)	381 (355–407)	194 (184–204)	104 (99–108)
*p* het		0.94	0.31	0.16	0.51	0.63	0.66	0.10	0.09
Biopsy diagnosis									
Benign non-proliferative	70	119 (111–126)	0.58 (0.26–0.91)	310 (277–343)	3425 (3277–3573)	137 (128–145)	401 (375–427)	180 (171–190)	107 (101–113)
Proliferative with/without atypia	97	125 (118–133)	1.16 (0.66–1.66)	353 (317–389)	3467 (3329–3605)	128 (123–134)	382 (363–400)	189 (181–197)	108 (104–112)
In situ/invasive	26	119 (102–137)	0.46 (0.03–0.88)	321 (264–377)	3502 (3181–3823)	124 (113–136)	361 (324–398)	198 (183–213)	107 (100–115)
*p* trend (*p* het)		0.65 (0.49)	0.73 (0.07)	0.38 (0.21)	0.60 (0.87)	*0.04* (0.10)	0.07 (0.19)	*0.04* (0.13)	0.93 (0.97)

*CI* confidence interval, *IGF* insulin-like growth factor, *IGFBP* IGF binding protein

^1^All IGF measures were log-transformed

*p* values < 0.05 are in italics

**Table 3:** Relationship of circulating unadjusted geometric mean concentrations of IGF-I and IGFP-1–7 (ng/mL) with select participant characteristics in postmenopausal women

Characteristics	*N*	IGF-I	IGFBP-1	IGFBP-2	IGFBP-3	IGFBP-4	IGFBP-5	IGFBP-6	IGFBP-7
		Mean^1^ (95% CI)	Mean (95% CI)	Mean (95% CI)	Mean (95% CI)	Mean (95% CI)	Mean (95% CI)	Mean (95% CI)	Mean (95% CI)
Age at biopsy (years)									
<55	30	105 (94–116)	0.66 (0.09–1.23)	335 (271–398)	3563 (3248–3878)	150 (135–165)	388 (357–420)	184 (166–201)	113 (106–121)
55–59	38	111 (101–121)	0.40 (0.09–0.72)	333 (275–392)	3578 (3370–3787)	159 (145–172)	416 (384–447)	164 (150–177)	115 (110–121)
≥ 60	35	92 (81–103)	0.94 (0.42–1.63)	333 (278–388)	3340 (3027–3652)	159 (147–170)	377 (348–405)	181 (168–194)	121 (114–128)
*p* trend (*p* het)		0.67 (*0.03*)	0.51 (0.32)	0.97 (0.99)	0.26 (0.40)	0.38 (0.61)	0.54 (0.18)	0.87 (0.10)	0.08 (0.20)
Education									
≤ High school grad/GED	22	108 (93–124)	3.13 (1.88-4.37)	287 (221–353)	3694 (3383–4005)	167 (147–186)	398 (356–440)	173 (155–192)	119 (107–131)
Some college/tech school	19	105 (93–118)	2.79 (1.81-3.78)	314 (252–375)	3389 (3031–3748)	151 (136–166)	415 (379–451)	191 (165–216)	116 (107–126)
≥ College graduate	62	100 (92–108)	3.42 (2.68-4.15)	359 (311–406)	3453 (3236–3670)	154 (145–164)	386 (363–409)	171 (162–181)	116 (113–120)
*p* trend (*p* het)		0.31 (0.59)	0.56 (0.67)	0.07 (0.20)	0.33 (0.44)	0.29 (0.39)	0.46 (0.50)	0.58 (0.25)	0.65 (0.88)
Body mass index (kg/m2)									
< 25	41	104 (95–112)	1.62 (0.61–2.63)	446 (397–494)	3343 (3154–3532)	141 (130–153)	396 (371–421)	166 (154–177)	114 (110–119)
25.0–29.9	31	103 (91–114)	0.83 (0.20–1.47)	323 (263–383)	3631 (3328–3934)	168 (153–182)	388 (354–423)	179 (164–193)	112 (106–118)
≥ 30	31	101 (87–115)	0.13 (0.03–0.23)	235 (194–276)	3554 (3183–3926)	166 (155–177)	398 (362–433)	185 (166–203)	125 (117–133)
*p* trend (*p* het)		0.78 (0.96)	*< 0.0001 (< .0001)*	*< 0.0001 (< .0001)*	0.25 (0.30)	*0.005 (0.004)*	0.95 (0.92)	0.06 (0.16)	*0.03 (0.02)*
Age at menarche (years)									
≤ 12	42	105 (97–112)	0.44 (0.11–0.76)	317 (262–371)	3587 (3352–3821)	157 (146–168)	389 (363–416)	180 (166–194)	114 (109–119)
13	34	99 (86–111)	0.89 (0.17–1.61)	374 (311–436)	3365 (3055–3675)	149 (136–161)	392 (360–423)	176 (164–188)	120 (114–126)
≥ 14	26	109 (96–122)	0.63 (0.09–1.17)	315 (256–374)	3558 (3259–3858)	163 (144–181)	409 (371–447)	166 (147–184)	117 (108–126)
*p* trend (*p* het)		0.72 (0.46)	0.45 (0.44)	0.87 (0.32)	0.76 (0.48)	0.71 (0.38)	0.43 (0.68)	0.19 (0.41)	0.40 (0.44)
Age at first birth (years)									
Nulliparous/ ≥ 30 years	37	108 (98–118)	0.54 (0.11–0.97)	359 (300–418)	3588 (3351–3825)	151 (137–165)	388 (357–419)	178 (163–193)	116 (111–122)
< 30 years	66	100 (92–108)	0.67 (0.29–1.05)	320 (279–361)	3438 (3225–3650)	159 (150–168)	397 (376–419)	174 (163–184)	117 (112–122)
*p* het		0.24	0.64	0.29	0.39	0.30	0.63	0.64	0.82
Parity									
Nulliparous (0)	22	103 (89–116)	0.43 (− 0.02–0.89)	326 (253–398)	3542 (3197–3888)	160 (140–180)	365 (334–395)	187 (167–208)	118 (110–125)
1	20	105 (90–119)	0.71 (− 0.09–1.52)	326 (244–408)	3468 (3151–3785)	152 (135–168)	417 (375–458)	171 (151–192)	113 (107–119)
2	42	99 (88–110)	0.76 (0.24–1.29)	338 (288–389)	3445 (3150–3739)	163 (153–173)	393 (366–420)	179 (168–190)	121 (114–128)
≥ 3	19	110 (99–120)	0.51 (−0.05–1.06)	341 (255–428)	3559 (3278–3841)	142 (125–160)	408 (356–460)	158 (138–178)	111 (104–118)
*p* trend (*p* het)		0.78 (0.71)	0.72 (0.80)	0.72 (0.99)	0.95 (0.95)	0.40 (0.23)	0.23 (0.27)	0.09 (0.15)	0.71 (0.17)
Characteristics	*N*	IGF-I	IGFBP-1	IGFBP-2	IGFBP-3	IGFBP-4	IGFBP-5	IGFBP-6	IGFBP-7
		Mean (95% CI)	Mean (95% CI)	Mean (95% CI)	Mean (95% CI)	Mean (95% CI)	Mean (95% CI)	Mean (95% CI)	Mean (95% CI)
Oral contraceptive use									
Never	15	90 (77–103)	1.14 (−0.04–2.32)	291 (230–352)	3214 (2799–3630)	164 (138–190)	414 (374–454)	175 (151–199)	117 (109–126)
Former	88	105 (98–112)	0.56 (0.27–0.84)	342 (303–380)	3540 (3367–3714)	155 (147–163)	391 (371–410)	175 (166–184)	117 (113–121)
*p* het		0.08	0.29	0.28	0.15	0.41	0.38	0.99	0.89
Menopausal hormone use									
Never	67	101 (93–110)	0.65 (0.28–1.01)	329 (287–372)	3464 (3270–3659)	159 (149–168)	392 (371–413)	174 (164–184)	116 (111–121)
Former	36	105 (96–115)	0.57 (0.11–1.03)	342 (287–398)	3541 (3252–3830)	152 (139–165)	398 (365–431)	177 (161–193)	118 (113–124)
*p* het		0.55	0.81	0.72	0.66	0.40	0.75	0.79	0.60
Family history of breast cancer in a first-degree relative									
None	75	103 (97–110)	0.53 (0.24–0.83)	333 (295–371)	3490 (3321–3660)	155 (146–164)	391 (371–412)	172 (162–182)	117 (112–121)
1 or more	27	102 (86–118)	0.90 (0.13–1.67)	344 (270–418)	3469 (3073–3865)	160 (145–174)	404 (366–441)	184 (167–200)	117 (111–123)
*p* het		0.83	0.33	0.78	0.91	0.59	0.55	0.26	0.88
Age at menopause									
< 45	17	95 (77–113)	0.91 (− 0.01–1.84)	287 (208–367)	3389 (2996–3783)	168 (143–193)	411 (378–444)	181 (158–203)	118 (111–126)
45–49	26	114 (103–126)	0.40 (0.02–0.79)	305 (245–365)	3585 (3257–3913)	153 (140–166)	415 (377–453)	164 (148–180)	119 (109–130)
≥ 50	48	105 (97–112)	0.57 (0.18–0.96)	351 (300–402)	3607 (3411–3803)	153 (142–165)	380 (353–407)	180 (168–193)	115 (111–120)
*p* trend (*p* het)		0.53 (0.11)	0.67 (0.54)	0.14 (0.32)	0.36 (0.58)	0.28 (0.42)	0.14 (0.23)	0.70 (0.28)	0.48 (0.69)
Cigarette smoking									
Never	37	103 (91–116)	0.68 (0.16–1.21)	357 (296–418)	3491 (3144–3838)	157 (145–168)	387 (358–416)	184 (168–201)	119 (113–124)
Former	47	105 (97–114)	0.40 (0.12–0.68)	296 (250–342)	3546 (3371–3722)	155 (144–167)	418 (391–444)	174 (164–184)	117 (112–122)
Current	11	89 (71–108)	2.60 (−0.06–5.25)	382 (297–466)	3167 (2789–3544)	178 (151–204)	355 (307–402)	149 (125–173)	113 (93–133)
*p* trend (*p* het)		0.39 (0.31)	0.45 (0.06)	0.66 (0.16)	0.45 (0.36)	0.29 (0.25)	0.87 (0.07)	*0.02 (0.04)*	0.43 (0.70)
Breast biopsy prior to enrollment									
Never	62	102 (94–109)	0.67 (0.26–1.08)	344 (300–387)	3429 (3251–3607)	158 (148–168)	404 (384–425)	175 (163–186)	115 (112–119)
Ever	39	105 (93–117)	0.50 (0.13–0.87)	313 (258–369)	3610 (3287–3933)	156 (144–167)	379 (346–412)	177 (163–190)	119 (112–127)
*p* het		0.66	0.55	0.39	0.30	0.76	0.18	0.84	0.32
Biopsy diagnosis									
Benign non-proliferative	31	103 (93–113)	0.94 (0.19–1.68)	369 (313–426)	3556 (3282–3830)	152 (137–166)	409 (379–439)	169 (152–186)	114 (107–121)
Proliferative with/without atypia	47	103 (92–114)	0.47 (0.13–0.80)	307 (257–356)	3499 (3237–3760)	163 (151–175)	381 (353–409)	180 (168–192)	121 (115–127)
In-situ/invasive	25	103 (91–114 s)	0.63 (0.05–1.21)	345 (271–418)	3397 (3097–3698)	149 (136–162)	401 (368–433)	175 (159–191)	112 (107–117)
*p* trend (*p* het)		0.95 (0.99)	0.49 (0.46)	0.55 (0.29)	0.49 (0.78)	0.87 (0.28)	0.67 (0.39)	0.57 (0.56)	0.75 (0.12)

*CI* confidence interval, *IGF* insulin-like growth factor, *IGFBP* IGF binding protein

^1^IGF-measures were log-transformed

*p* values < 0.05 are in italics

#### Age

Among premenopausal women, IGF-I tended to be inversely associated with age (*p* trend = 0.005), whereas IGFBP-6 was positively associated with age (*p* trend = 0.009) (Table 2). In contrast, none of the IGF measures demonstrated statistically significant trends with age among postmenopausal women (Table 3).

#### Education

Among premenopausal women, education was positively associated with IGFBP-5 (*p* trend = 0.001) (Table 2). Among postmenopausal women, IGF measures were not significantly associated with education (Table 3).

#### Body mass index

Among premenopausal women, concentrations of IGFBP-1 and IGFBP-2 decreased with increasing BMI (*p* trend ≤ 0.0001), whereas IGFBP-3 increased with increasing BMI (*p* trend = 0.007) (Table 2). Similarly, among postmenopausal women, we also observed inverse associations between IGFBP-1 and IGFBP-2 with BMI (*p* trend ≤ 0.0001) (Table 3). However, no statistically significant associations with BMI were observed for IGFBP-3. In addition, among postmenopausal women, concentrations of IGFBP-4 and IGFBP-7 were positively associated with BMI (*p* trend = 0.005 and 0.03, respectively).

#### Age at first birth

Among premenopausal women, IGFBP-3 concentration was higher in women with age at first birth < 30 years compared to women who were nulliparous or whose age at first birth was ≥ 30 years (*p* het = 0.007) (Table 2). None of the remaining IGF measures were significantly associated with age at first birth among pre- or postmenopausal women (Tables 2 and 3).

#### Cigarette smoking

Among premenopausal women, IGFBP-5 concentration was lower in current and former as compared to never smokers (*p* trend = 0.005) (Table 2). Though significant heterogeneity in IGFBP-4 (*p* het = 0.01) level was also observed by smoking status, no clear trend was observed. Among postmenopausal women, IGFBP-6 decreased across former and current smoking groups (*p* trend = 0.02) (Table 3).

Other participant characteristics such as age at menarche, education, parity, oral contraceptive use, menopausal hormone use, family history of breast cancer in a first-degree relative, age at menopause, breast biopsy prior to enrollment, and biopsy diagnosis were not significantly associated with the IGF measures in either pre- or postmenopausal women.

### Relationship of circulating IGF-I and IGFBPs with mammographic density measures

Age, BMI, and BMI^2^-adjusted associations of IGF measures with mammographic density measures—percent dense volume/area, absolute dense volume/area, and non-dense volume/area—for pre- and postmenopausal women are presented in Tables 4 and 5, respectively.

**Table 4:** Age- and BMI-adjusted^2^ linear regression estimates for associations between IGF measures (pg/mL) and mammographic density measures, premenopausal women (*N* = 189)

IGF measures	% MD-V^1^		% MD-A		Absolute MD-V		Absolute MD-A		Non-dense volume		Non-dense area	
	*β*	*p* ^4^	*β*	*p*	*β*	*p*	*β*	*p*	*β*	*p*	*β*	*p*
IGF-I	0.11	0.97	− 1.29	0.69	− 0.39	0.95	− 0.42	0.92	− 10.33	0.33	− 1.99	0.65
IGFBP-1^3^	17.19	0.51	21.93	0.51	− 5.38	0.94	18.15	0.66	− 133.91	0.22	− 34.22	0.46
IGFBP-2	1.49	*0.02*	1.55	0.05	0.35	0.83	1.48	0.13	− 6.56	*0.01*	− 1.74	0.11
IGFBP-3	− 0.20	0.15	− 0.34	0.05	0.03	0.93	− 0.24	0.28	0.57	0.33	0.33	0.18
IGFBP-4	− 7.19	*0.01*	− 5.08	0.18	− 5.55	0.46	− 4.13	0.38	25.06	*0.04*	5.09	0.33
IGFBP-5	− 0.50	0.50	0.37	0.69	− 0.37	0.84	0.45	0.70	0.48	0.88	− 1.21	0.35
IGFBP-6	0.89	0.71	2.37	0.44	9.34	0.12	5.26	0.17	1.73	0.86	0.34	0.94
IGFBP-7	− 0.80	0.83	− 0.89	0.85	− 3.52	0.71	− 2.59	0.66	− 0.90	0.95	− 1.59	0.81
IGF-I:IGFBP-1	< 0.001	0.91	< − 0.001	0.62	< − 0.001	0.67	< − 0.001	0.43	< − 0.001	0.61	< − 0.001	0.71
IGF-I:IGFBP-2	− 0.17	0.07	− 0.20	0.10	−0.17	0.47	−0.19	0.21	0.39	0.33	0.10	0.56
IGF-I:IGFBP-3	3.31	0.28	3.29	0.40	0.003	0.99	3.27	0.50	− 22.38	0.08	− 7.38	0.17
IGF-I:IGFBP-4	0.05	0.46	− 0.03	0.73	0.05	0.80	− 0.01	0.92	− 0.40	0.18	− 0.04	0.74
IGF-I:IGFBP-5	− 0.02	0.93	− 0.32	0.25	0.01	0.99	− 0.23	0.51	− 0.37	0.69	0.36	0.36
IGF-I:IGFBP-6	− 0.03	0.83	− 0.19	0.29	− 0.37	0.29	− 0.27	0.23	− 0.64	0.28	− 0.11	0.64
IGF-I:IGFBP-7	− 0.04	0.54	− 0.07	0.41	− 0.05	0.78	− 0.04	0.70	− 0.08	0.76	0.01	0.93
IGF-I:Total IGFBP	2.99	0.46	1.35	0.80	0.20	0.98	1.76	0.78	− 24.14	0.16	− 6.35	0.37

*IGF* insulin-like growth factor, *IGFBP* IGF binding protein, *MD-V* mammographic density-volume, *MD-A* MD-area

^1^Mammographic density measures were square-root transformed

^2^All models were adjusted for age, BMI, and BMI^2^ in the model. Excluded women with BMI > 45 (N=4)

^3^For IGFBP-1, *N* = 91 samples were below the lower limit of detection (LLOD) and were reassigned as ½ LLOD (0.025)

^4^*p* values < 0.05 are in italics

**Table 5:** Age- and BMI-adjusted linear^2^ regression estimates for associations between IGF measures (pg/mL) and mammographic density measures, postmenopausal women (*N* = 101)

IGF measures	% MD-V^1^		% MD-A		Absolute MD-V		Absolute MD-A		Non-Dense Volume		Non-Dense Area	
	*β*	*p* ^4^	*β*	*p*	*β*	*p*	*β*	*p*	*β*	*p*	*β*	*p*
IGF-I	− 0.55	0.89	1.97	0.70	− 6.57	0.49	3.10	0.64	− 5.95	0.77	− 5.26	0.48
IGFBP-1^3^	50.06	0.15	− 49.56	0.28	− 84.48	0.34	− 77.58	0.20	− 418.94	*0.02*	− 8.09	0.91
IGFBP-2	2.04	*0.003*	− 0.01	0.99	− 1.29	0.47	− 0.89	0.47	− 13.13	*0.0003*	− 1.33	0.33
IGFBP-3	− 0.13	0.37	0.21	0.28	− 0.51	0.16	0.32	0.20	0.06	0.93	− 0.26	0.36
IGFBP-4	− 1.72	0.55	− 2.22	0.56	2.74	0.70	− 1.03	0.84	12.02	0.43	8.42	0.13
IGFBP-5	− 0.74	0.53	− 0.10	0.95	− 0.36	0.91	0.80	0.70	2.67	0.67	− 0.29	0.90
IGFBP-6	− 3.08	0.21	− 4.67	0.15	2.20	0.72	− 3.89	0.37	26.46	*0.04*	11.89	*0.01*
IGFBP-7	3.56	0.53	− 5.21	0.49	14.32	0.32	− 9.46	0.34	− 1.77	0.95	8.43	0.45
IGF-I:IGFBP-1	< − 0.001	*0.04*	< 0.001	0.70	< − 0.001	0.85	< 0.001	0.33	0.0002	*0.03*	< 0.001	0.75
IGF-I:IGFBP-2	− 0.16	0.06	0.12	0.30	− 0.08	0.72	0.22	0.16	0.88	0.06	− 0.03	0.86
IGF-I:IGFBP-3	3.07	0.53	− 5.59	0.39	4.70	0.70	− 8.64	0.31	− 13.09	0.61	0.61	0.95
IGF-I:IGFBP-4	0.01	0.96	0.14	0.41	− 0.14	0.67	0.19	0.38	− 0.11	0.87	− 0.25	0.31
IGF-I:IGFBP-5	0.12	0.71	0.24	0.57	− 0.15	0.86	0.26	0.65	− 0.58	0.73	− 0.23	0.71
IGF-I:IGFBP-6	0.11	0.44	0.19	0.32	− 0.32	0.38	0.16	0.53	− 1.26	0.10	− 0.62	*0.03*
IGF-I:IGFBP-7	− 0.07	0.54	0.11	0.45	− 0.20	0.45	0.22	0.24	0.11	0.84	− 0.11	0.59
IGF-I:Total IGFBP	0.72	0.91	− 2.95	0.72	1.37	0.93	− 4.15	0.71	− 4.38	0.90	− 2.71	0.83

*IGF* insulin-like growth factor, *IGFBP* IGF binding protein, *MD-V* mammographic density-volume, *MD-A* MD-area

^1^Mammographic density measures were square-root transformed

^2^All models were adjusted for age, BMI, and BMI^2^ in the model. Excluded women with BMI > 45 (N=2)

^3^For IGFBP-1, *N* = 91 samples were below the lower limit of detection (LLOD) and were reassigned as ½ LLOD (0.025)

^4^*p* values < 0.05 are in italics

#### Mammographic measures of percent dense volume/area

Among premenopausal women, IGFBP-2 was positively associated with percent MD-V (*β* = 1.49, *p* = 0.02) and percent MD-A (*β* = 1.55, *p* = 0.05) (Table 4). IGFBP-4 was inversely associated with percent MD-V (*β* = − 7.19, *p* = 0.01). The ratio of IGF-I:IGFBP-2 was also borderline inversely associated with percent MD-V (*β* = − 0.17, *p* value = 0.07). In contrast, the ratio of IGF-I:IGFBP-3 was borderline positively associated with percent MD-V (*β* = 5.46, *p* = 0.08) before the inclusion of a quadratic term for BMI as an adjustment factor (Additional file 1: Table S4); however, after fully adjusting for BMI, neither IGF-I, IGFBP-3, nor their ratio was associated with MD (Table 4).

Among postmenopausal women, IGFBP-2 was positively associated with percent MD-V (*β* = 2.04, *p* = 0.003), but other IGF measures were not significantly associated with percent density measures (Table 5). In *post hoc* analyses, the patterns of association between IGFBP-2 and MD measures were consistent in both pre- and postmenopausal women even after adjustment by the most abundant binding protein, IGFBP-3 (data not shown).

Effect modification of the association between IGFBP-2 and MD was also considered as a *post hoc* sensitivity analysis because we observed strong main effects for the relation of both BMI and MD with IGFBP-2, a finding that was not observed for the other IGF measures. Results from BMI-stratified (lean: < 25 kg/m^2^; overweight: 25–29.9 kg/m^2^; obese: ≥30 kg/m^2^) analyses are shown in (Table 6). We observed borderline significant positive associations between IGFBP-2 and percent MD-V among premenopausal women who were lean (*β* = 1.32, *p* = 0.07) and over-weight (*β* = 2.32, *p* = 0.06), but not obese (*β* = − 0.58, *p* = 0.79); *p* interaction = 0.57. Among postmenopausal women, the positive relationships between IGFBP-2 and percent MD-V and MD-A were driven primarily those observed in lean women (MD-V: *β* = 5.32, *p* = 0.0002; MD-A: *β* = 3.24, *p* = 0.07); *p* interaction ≤ 0.03.

**Table 6:** Age- and BMI-adjusted linear regression estimates for relationships between IGFBP-2 and % MD measures in premenopausal and postmenopausal women, stratified by BMI

	BMI						
	< 25 kg/m^2^ (*N* = 97)		25–29.9 kg/m^2^ (*N* = 48)		≥ 30 kg/m^2^ (*N* = 48)		
MD measure	*β*	*p* ^1^	*β*	*p*	*β*	*p*	*p* -int
*Premenopausal women (N = 193)*							
% MD-V	1.32	0.07	2.32	0.06	−0.58	0.79	0.57
% MD-A	1.24	0.20	2.31	0.13	0.10	0.97	0.55
	BMI						
*Postmenopausal women (N = 103)*	< 25 kg/m^2^ (*N* = 41)		25–29.9 kg/m^2^ (*N* = 31)		≥ 30 kg/m^2^ (*N* = 31)		
% MD-V	5.32	*0.0002*	0.81	0.24	− 0.29	0.85	*0.0003*
% MD-A	3.24	0.07	− 1.35	0.28	− 1.75	0.34	*0.03*

*p* -int *p* interaction, *BMI* body mass index, *MD-V* mammographic density-volume, *MD-A* MD-area

^1^P-values <0.05 are in italics

#### Mammographic measure of absolute dense and non-dense volume/area

IGF measures were not associated with absolute MD-V or MD-A in either pre- or postmenopausal women (Tables 4 and 5). However, numerous IGF analytes were associated with non-dense volume and area measures. For example, among premenopausal women, IGFBP-2 was statistically significantly inversely associated with non-dense volume and IGFBP-4 was statistically significantly positively associated with non-dense volume (Table 4). Among postmenopausal women, IGFBP-1 and IGFBP-2 were inversely associated with non-dense volume (Table 5). IGFBP-6 was positively associated with both non-dense volume and area, and IGF-I:IGFBP-1 was positively associated with non-dense volume (Table 5). IGF-I:IGFBP-6 was inversely associated with non-dense area (Table 5).

#### Factor analysis

**Fig. 2:**
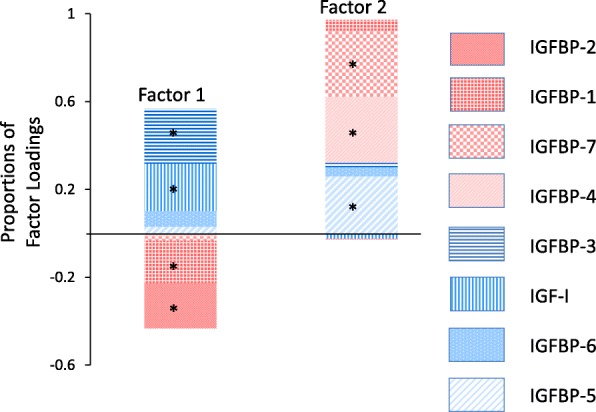
Results from factor analysis depicting the relative contribution of each insulin-like growth factor (IGF) measure to two independent resulting factors in BREAST Stamp Project participants. IGF, insulin-like growth factor; IGFBP, IGF binding protein; factor 1 was positively correlated with IGF-I and IGFBP-3 and inversely correlated with IGFBP-1 and IGFBP-2; factor 2 was positively correlated with IGFBP-4, IGFBP-5, and IGFBP-7. The proportion of each analyte was determined as the ratio of the factor loading to the sum of absolute values of factor loadings. *IGF measures that were significantly involved in each factor. Factor loading *p* < 0.05

Factor analysis of IGF measures revealed 2 independent factors (Additional file 1: Table S6 and Fig. 2). Main contributors to factor 1 were IGFBP-3, IGF-I, IGFBP-1, and IGFBP-2. IGF-I and IGFBP-3 positively contributed to factor 1, and IGFBP-1 and IGFBP-2 negatively contributed to factor 1. IGFBP-4, IGFBP-5, and IGFBP-7 positively contributed to factor 2. The variance in the IGF measures explained by factor 1 was 53.04%, 53.81%, and 55.93% in overall, premenopausal, and postmenopausal women, respectively. The variance explained by factor 2 was 46.96%, 46.19%, and 44.07% in overall, premenopausal, and postmenopausal women. The composition of the factors was similar in overall women and each menopausal group even though the levels of IGF measures varied by menopausal status. When we conducted factor analysis using 3 factors, the contribution of each of the IGF proteins to the factors was similar to the model using 2 factors; thus, results are presented for 2 factors.

Table 7 shows the age, BMI, and BMI^2^-adjusted linear regression model estimates for the association between quintiles of factor 1 and 2 with MD measures. Among premenopausal women, borderline inverse associations of high levels of factor 1 with lower percent MD-V (*β* = − 0.13, *p* value = 0.08) and percent MD-A (*β* = − 0.18, *p* value = 0.05) were observed. Among postmenopausal women, factor 1 was also inversely associated with percent MD-V (*β* = − 0.19, *p* value = 0.02). Factor 1 was also positively associated with non-dense volume (*β* = 0.91, *p* value = 0.04). In both pre- and postmenopausal women, factor 2 was not significantly associated with MD measures.

**Table 7:** Age- and BMI-adjusted^1^ linear regression estimates for the relation of IGF factors (in quintiles) with mammographic density measures

	% MD-V^1^		% MD-A		Absolute MD-V		Absolute MD-A		Non-dense volume		Non-dense area	
	*β*	*p* ^3^	*β*	*p*	*β*	*p*	*β*	*p*	*β*	*p*	*β*	*p*
Premenopausal women												
Factor 1^2^	− 0.13	0.08	− 0.18	0.05	0.01	0.97	− 0.13	0.27	0.40	0.19	0.16	0.23
Factor 2	− 0.05	0.49	− 0.04	0.65	− 0.11	0.53	− 0.73	0.50	0.07	0.80	− 0.04	0.74
Postmenopausal women												
Factor 1	− 0.19	*0.02*	0.09	0.43	− 0.09	0.67	0.20	0.17	0.91	*0.04*	− 0.01	0.93
Factor 2	− 0.03	0.73	− 0.66	0.55	0.02	0.94	− 0.07	0.61	0.06	0.98	0.09	0.59

*MD-V* mammographic density-volume, *MD-A* MD-area

^1^All models were adjusted for age, BMI, and BMI^2^ in the model. Excluded women with BMI > 45

^2^Factor 1 was positively correlated with IGF-I and IGFBP-3 and inversely correlated with IGFBP-1 and IGFBP-2; factor 2 was positively correlated with IGFBP-4, IGFBP-5, and IGFBP-7. Quintiles’ cutpoints for factor 1 and factor 2 were created based on their distributions in premenopausal and postmenopausal women separately

^3^*p* values < 0.05 are in italics

## Discussion

In this study of women undergoing diagnostic breast biopsy, we undertook an analysis of circulating IGF-I and seven of its binding proteins to examine relationships with MD. We did not observe clear associations between IGF-I, IGFBP-3, and MD after BMI adjustment. While prior studies have largely focused on IGF-I and IGFBP-3, we identified novel positive associations between IGFBP-2 and percent MD that withstood rigorous adjustment for BMI, suggesting that IGFBP-2 may play a key role in MD. In concert with in vitro studies demonstrating a dual role of IGFBP-2 on breast tissue (i.e., promoting cell proliferation and inhibiting adipogenesis), our findings suggest that further studies investigating the role of IGFBP-2 in breast tissue composition and the carcinogenic process are warranted.

Prior work indicates that the relation of IGF-I:IGFBP-3 with MD differs by menopausal status, with most studies demonstrating positive associations for premenopausal women, but null associations for postmenopausal women [15–19, 21–24]. We too observed similar associations by menopausal status that were attenuated upon adjusting fully for BMI. In addition to the widely studied IGF proteins (IGF-I and IGFBP-3), this study assessed for the first time the relationship of other IGF binding proteins with MD.

Interestingly, we identified IGFBP-2 as being positively associated with percent MD among all women, a finding which persisted after adjustment for BMI, despite a strong and inverse relation of BMI with both IGFBP-2 [26] and percent MD [39]. When we stratified analyses by BMI, we found that the observed positive association between IGFBP-2 and percent MD was most apparent among women with lower BMI, particularly among postmenopausal women. Though we cannot rule out the possibility that the association between IGFBP-2 and percent MD may be due to residual confounding by adiposity, both laboratory and epidemiologic studies have suggested an important role of IGFBP-2 in breast cancer etiology. Prior epidemiologic studies have reported inverse associations between IGFBP-2 and risk of atypical hyperplasia [26] and postmenopausal breast cancer [27]. Other studies have reported null findings for circulating IGFBP-2-associated breast cancer risk relationships [25, 49, 50]. Experimental studies have shown that IGFBP-2 can either inhibit or promote tumorigenesis [30, 51]. IGFBP-2 may inhibit tumorigenesis by inhibiting adipogenesis [52] and promoting apoptosis in an IGF-independent mechanism [53]. Conversely, IGFBP-2 is thought to induce tumorigenesis by promoting cell proliferation and invasion through suppression of PTEN activation and prolonged activity of PI3K [30, 51, 54] . Thus, with IGFBP-2’s hypothesized dual effect on breast tissue, it is biologically plausible that the proliferative effect of IGFBP-2 on breast epithelium, along with its inhibition of preadipocyte differentiation into mature adipocytes [55], may be reflected radiologically in elevated breast density.

The relationship between IGF proteins and MD measures varied by menopausal status in this study. As several of the IGF proteins as well as the MD measures vary by age and menopausal status, this finding is not necessarily surprising. Further, differences in interactions between circulating hormones, IGF proteins, and the tissue microenvironment [56, 57] may contribute to observed differences by menopausal status. For example, estrogen, a strong breast cancer risk factor, both regulates and is influenced by the IGF family [58]. To better understand the relationship between IGF proteins and MD, future larger studies assessing interrelationships between IGF, hormones, and breast tissue composition in pre-, peri-, and postmenopausal women will be important.

In this study, both volumetric and area mammographic density measures were assessed, and IGF associations with MD-A and MD-V were largely consistent. We have previously shown volumetric and area measures of MD to be highly correlated [39]. We found in multivariate analyses that IGF proteins appeared to be associated with percent MD-V/MD-A, but not absolute dense volume/area measures. Interestingly, we also observed that many of the IGFBPs were inversely associated with non-dense area, suggesting that IGFBPs may also be important correlates of adipose tissue composition in the breast. Indeed, in vitro studies have suggested a role for several IGFBPs in adipogenesis [59]. The role of breast adipose tissue reflected by the radiologic non-dense area in breast cancer development is not well understood [60, 61]. Future mechanistic studies are warranted to study interrelationships between adipocyte biology and endogenous growth factors like IGFBP, with stromal, epithelial, and adipose tissues that are reflected radiologically in MD.

A major strength of this study is that we investigated the relationship of multiple IGFBPs, in addition to the widely studied IGF-I and IGFBP-3, with quantitative, reliable measures of MD. IGF assays used were also highly reliable, with the IGF proteins demonstrating several expected relationships with breast cancer risk factors [14, 62–67]. For example, we found that IGF-I was inversely associated with age, likely due to declines in growth hormone secretion in with aging [63, 66–70]. As expected, IGFBP-2 was inversely associated with BMI [26]. In contrast to published studies [14, 64, 65, 67], we did not see an association of IGF-I with BMI, though some studies report that only the most extreme BMI levels are associated with the lowest IGF-I, whereas others hypothesize that IGF-I increases with body weight until a threshold is reached and then it triggers the feedback loop suppressing GH secretion. A non-linear U-shaped relationship between BMI and IGF-I has also been reported [71, 72]. We possibly did not see the association due to the low prevalence of obesity in our study population. In examining published literature on serums IGF levels in pre- and postmenopausal women, we found that the levels of IGF-I, IGFBP-3, and their ratio that we observed were within comparable ranges to those reported by the Nurses’ Health Study [19]. Likewise, levels of IGFBP-2 in our study were comparable with those previously reported from the nested case-control study within Women’s Health Initiative Clinical Trial [26] and within a general community healthcare plan setting [27], lending external validity to our results.

A limitation of this study is that the circulating IGF proteins were measured in a single serum sample, which may not be an adequate representation of future IGF levels within a woman. However, a stability study of IGF-I and IGFBP-3 among premenopausal women in NHS-II demonstrated that the 3-year intraclass correlation coefficient was 0.70 for IGF-I and 0.74 for IGFBP-3 [73]. Thus, a single measurement of IGF-I and BP-3 may be representative for at least 3 years. For other IGF proteins, stability studies should be carried out in the future. In our study, a high proportion of IGFBP-1 (*N* = 91) was below the LLOD, perhaps precluding our ability to detect an association between IGFBP-1 and MD. Though alcohol drinking has been associated with MD [74–78], breast cancer risk [79], and IGF measures [80], information on alcohol consumption was not collected as part of this study. Our study also had a relatively small sample size, particularly upon stratification by menopausal status; larger studies are needed to validate our observed associations between IGF and MD measures. In addition, we measured circulating IGF proteins, and systemic IGFs may differ from those regulating tissue levels [81, 82]. Nevertheless, there is evidence demonstrating that local tissue expression of the IGF proteins may influence the systemic expression of IGF proteins [83, 84].

## Conclusions

We demonstrated a novel positive association between circulating IGFBP-2 and percent MD, particularly among women with lower BMI. These results contribute to the rationale for evaluating relationships between serum IGFs, their multiple binding proteins, and breast cancer risk factors, including MD, to advance our understanding of the IGF proteins in breast cancer etiology.

## Additional file

Additional file 1: **Table S1.** Selected characteristics of BREAST Stamp Project participants stratified by menopausal status. **Table S2.** Correlation among IGF-measures, by menopausal status. **Table S3.** Distribution of circulating IGF-I and IGFBPs 1–7 (ng/mL) among BREAST Stamp Project participants whose biopsies yielded benign diagnoses, stratified by menopausal status. **Table S4.** Age- and BMI-adjusted^2^ linear regression estimates for associations between IGF measures (pg/mL) and mammographic density measures, premenopausal women (*N* = 193). **Table S5.** Age- and BMI-adjusted^2^ linear regression estimates for associations between IGF measures (pg/mL) and mammographic density measures, postmenopausal women (*N* = 103). **Table S6.** Factor loadings for IGF analytes, overall, and by menopausal status. **Table S7.** Age- and BMI-adjusted^1^ linear regression estimates for the relation of IGF factors (in quintiles) with mammographic density measures. (DOCX 54 kb)

## Data Availability

The dataset generated and/or analyzed during the current study is available from the corresponding author on request.

## Abbreviations

ANOVA: Analysis of variance
BMI: Body mass index
BREAST: Breast Radiology Evaluation and Study of Tissues
CLIA: Chemiluminescence immunoassay
ICC: Intraclass correlations
IGF: Insulin-like growth factor
IGFBPs: Insulin-like growth factor binding proteins
LLOD: Lower limit of detection
MD: Mammographic density
MD-A: Area density measure
MD-V: Volumetric density measure
NCI: National Cancer Institute
SXA: Single X-ray absorptiometry
UCSF: University of California at San Francisco
UVM: University of Vermont
