# Supplementary material for: Relationship of circulating insulin-like growth factor-I and binding proteins 1–7 with mammographic density among women undergoing image-guided diagnostic breast biopsy
Source: Breast Cancer Res. 2019 Jul 23;21:81. doi: 10.1186/s13058-019-1162-8 (PMC6651938; doi:10.1186/s13058-019-1162-8)
Supplement: Supplementary file 1 — Table S1. Selected characteristics of BREAST Stamp Project participants stratified by menopausal status. Table S2. Correlation among IGF-measures, by menopausal status. Table S3. Distribution of circulating IGF-I and IGFBPs 1–7 (ng/mL) among BREAST Stamp Project participants whose biopsies yielded benign diagnoses, stratified by menopausal status. Table S4. Age- and BMI-adjusted2 linear regression estimates for associations between IGF measures (pg/mL) and mammographic density measures, premenopausal women (N = 193). Table S5. Age- and BMI-adjusted2 linear regression estimates for associations between IGF measures (pg/mL) and mammographic density measures, postmenopausal women (N = 103). Table S6. Factor loadings for IGF analytes, overall, and by menopausal status. Table S7. Age- and BMI-adjusted1 linear regression estimates for the relation of IGF factors (in quintiles) with mammographic density measures. (DOCX 54 kb) [file 13058_2019_1162_MOESM1_ESM.docx]

Table S1. Selected characteristics BREAST Stamp Project participants stratified by menopausal status

| **Characteristics** | **Premenopausal (N=193)** | |  | **Postmenopausal (N=103)** | |
| --- | --- | --- | --- | --- | --- |
|  | **N** | **%** |  | **N** | **%** |
| *Age at biopsy among premenopausal women (years)* |  |  |  |  |  |
| <45 | 56 | 29.0 |  | NA | NA |
| 45-49 | 83 | 43.0 |  | NA | NA |
| ≥50 | 54 | 28.0 |  | NA | NA |
| *Age at biopsy among postmenopausal women (years)* |  |  |  |  |  |
| >54 | NA | NA |  | 30 | 29.1 |
| 55-59 | NA | NA |  | 38 | 36.9 |
| ≥60 | NA | NA |  | 35 | 34.0 |
| *Race and ethnicity* |  |  |  |  |  |
| White, Non-Hispanic | 185 | 95.9 |  | 95 | 92.2 |
| Other | 8 | 4.2 |  | 8 | 7.8 |
| *Education* |  |  |  |  |  |
| *≤ High school grad/GED* | 27 | 14.0 |  | 22 | 21.4 |
| *Some college/tech school* | 37 | 19.2 |  | 19 | 18.5 |
| *≥ College graduate* | 129 | 66.8 |  | 62 | 60.2 |
| *Body mass index (kg/m2)* |  |  |  |  |  |
| <25 | 97 | 50.3 |  | 41 | 39.8 |
| 25.0-29.9 | 48 | 24.9 |  | 31 | 30.1 |
| ≥30 | 48 | 24.9 |  | 31 | 30.1 |
| *Age at menarche (years)* |  |  |  |  |  |
| <=12 | 69 | 36.3 |  | 42 | 41.2 |
| 13 | 73 | 38.4 |  | 34 | 33.3 |
| ≥14 | 48 | 25.3 |  | 26 | 25.5 |
| Missing | 3 | ~ |  | 1 | ~ |
| *Age at first birth (years)* |  |  |  |  |  |
| Nulliparous/ 30+ years | 94 | 49.0 |  | 37 | 36.0 |
| <30 years | 98 | 51.0 |  | 66 | 64.1 |
| Missing | 1 | ~ |  | 0 | ~ |
| *Parity* |  |  |  |  |  |
| Nulliparous (0) | 45 | 23.3 |  | 22 | 21.4 |
| 1 | 20 | 10.4 |  | 20 | 19.4 |
| 2 | 82 | 42.3 |  | 42 | 40.8 |
| ≥3 | 46 | 23.8 |  | 19 | 18.5 |
| *Oral contraceptive use* |  |  |  |  |  |
| Never | 28 | 14.5 |  | 15 | 14.6 |
| Ever | 165 | 85.5 |  | 88 | 85.4 |
| *Menopausal hormone use* |  |  |  |  |  |
| *Never* | 169 | 88.5 |  | 67 | 65.0 |
| *Former* | 22 | 11.5 |  | 36 | 35.0 |
|  |  |  |  |  |  |
| **Characteristics** | **Premenopausal (N=193)** | |  | **Postmenopausal (N=103)** | |
|  | **N** | **%** |  | **N** | **%** |
| *Family history of breast cancer in a first degree relative* |  |  |  |  |  |
| None | 145 | 75.5 |  | 75 | 73.5 |
| 1 or more | 47 | 24.5 |  | 27 | 26.5 |
| Missing | 1 | ~ |  | 1 | ~ |
| *Age at menopause* |  |  |  |  |  |
| <45 | ~ | ~ |  | 17 | 18.7 |
| 45-49 | ~ | ~ |  | 26 | 28.6 |
| ≥50 | ~ | ~ |  | 48 | 52.8 |
| Missing | ~ | ~ |  | 12 | ~ |
| *Cigarette smoking* |  |  |  |  |  |
| Never | 100 | 54.1 |  | 37 | 39.0 |
| Former | 65 | 35.1 |  | 47 | 50.0 |
| Current | 20 | 10.8 |  | 11 | 11.6 |
| Missing | 8 | ~ |  | 8 | ~ |
| *Breast Biopsy prior to enrollment* |  |  |  |  |  |
| Never | 130 | 67.4 |  | 62 | 61.4 |
| Ever | 63 | 32.6 |  | 39 | 38.6 |
| *Biopsy diagnosis* |  |  |  |  |  |
| Benign non-proliferative | 70 | 36.3 |  | 31 | 30.1 |
| Proliferative with/without atypia | 97 | 50.3 |  | 47 | 45.6 |
| In-situ / invasive | 26 | 13.5 |  | 25 | 24.3 |
| ***Mammographic Density (MD) Measures*** | **Mean** | **SD** |  | **Mean** | **SD** |
| % MD-Volume | 21.4 | 43.1 |  | 17.2 | 30.3 |
| % MD-Area | 20.2 | 31.9 |  | 16.7 | 20.1 |
| Absolute MD-Volume | 105 | 208 |  | 87.9 | 184 |
| Absolute MD-Area | 27 | 39.1 |  | 21.8 | 27.9s |
| Non-Dense Volume | 381 | 415 |  | 410 | 580 |
| Non-Dense Area | 75.5 | 105 |  | 83.4 | 143 |

Missing values were excluded from percentage calculations.

Table S2. Correlation among IGF-measures, by menopausal status

| **Premenopausal women untransformed IGF Measures; Spearman Correlation Coefficients (N=193)** | | | | | | | | |
| --- | --- | --- | --- | --- | --- | --- | --- | --- |
|  | **IGF-I** | **IGFBP-1** | **IGFBP-2** | **IGFBP-3** | **IGFBP-4** | **IGFBP-5** | **IGFBP-6** | **IGFBP-7** |
| **IGF-I** | 1.00 |  |  |  |  |  |  |  |
| **IGFBP-1** | -0.06 | 1.00 |  |  |  |  |  |  |
| **IGFBP-2** | -0.08 | **0.58** | 1.00 |  |  |  |  |  |
| **IGFBP-3** | **0.55** | -0.26 | **-0.38** | 1.00 |  |  |  |  |
| **IGFBP-4** | -0.07 | 0.15 | -0.06 | 0.10 | 1.00 |  |  |  |
| **IGFBP-5** | **0.26** | 0.11 | 0.08 | -0.09 | **0.35** | 1.00 |  |  |
| **IGFBP-6** | 0.15 | -0.02 | 0.11 | 0.23 | 0.03 | -0.14 | 1.00 |  |
| **IGFBP-7** | -0.06 | 0.09 | **0.20** | 0.04 | **0.30** | **0.15** | 0.27 | 1.00 |
| **Postmenopausal women untransformed IGF Measures; Spearman Correlation Coefficients (N=103)** | | | | | | | | |
|  | **IGF-I** | **IGFBP-1** | **IGFBP-2** | **IGFBP-3** | **IGFBP-4** | **IGFBP-5** | **IGFBP-6** | **IGFBP-7** |
| **IGF-I** | 1.00 |  |  |  |  |  |  |  |
| **IGFBP-1** | **-0.31** | 1.00 |  |  |  |  |  |  |
| **IGFBP-2** | -0.14 | **0.63** | 1.00 |  |  |  |  |  |
| **IGFBP-3** | **0.65** | **-0.33** | **-0.31** | 1.00 |  |  |  |  |
| **IGFBP-4** | -0.13 | -0.05 | **-0.21** | 0.07 | 1.00 |  |  |  |
| **IGFBP-5** | **0.25** | -0.11 | -0.17 | 0.14 | 0.18 | 1.00 |  |  |
| **IGFBP-6** | -0.04 | -0.08 | -0.03 | 0.06 | 0.17 | -0.19 | 1.00 |  |
| **IGFBP-7** | -0.09 | -0.03 | -0.07 | 0.03 | **0.34** | -0.04 | **0.44** | 1.00 |

*Correlations in bold font are statistically significant with P-values <0.05.

Table S3. Distribution of circulating IGF-I and IGFBPs 1-7 (ng/mL) among BREAST Stamp Project participants whose biopsies yielded benign diagnoses, stratified by menopausal status

| IGF Measure | Premenopausal (N= 167) | | |  | Postmenopausal (N= 78) | | |  | P-Value ^3^  (Wilcoxon) | |
| --- | --- | --- | --- | --- | --- | --- | --- | --- | --- | --- |
|  | Median^1^ | 10th Percentile | 90th Percentile |  | Median | 10th Percentile | 90th Percentile |  |  |  |
| IGF-I | 121 | 85.1 | 180.5 |  | 112 | 74 | 143 |  | **0.0004** |  |
| IGFBP-1^2^ | 2.5 | 0.02 | 6.67 |  | 1.95 | 0.02 | 7.63 |  | 0.40 |  |
| IGFBP-2 | 342 | 167 | 644 |  | 343 | 144 | 616 |  | 0.93 |  |
| IGFBP-3 | 3502 | 2715 | 4372 |  | 3559 | 2606 | 4508 |  | 0.20 |  |
| IGFBP-4 | 130 | 101 | 177 |  | 160 | 117 | 220 |  | **<0.0001** |  |
| IGFBP-5 | 392 | 280 | 511 |  | 400 | 286 | 532 |  | 0.70 |  |
| IGFBP-6 | 184 | 140 | 238 |  | 175 | 123 | 242 |  | 0.08 |  |
| IGFBP-7 | 105 | 89 | 134 |  | 118 | 98 | 143 |  | **<0.0001** |  |

IGF, insulin-like growth factor; IGFBP, IGF binding protein

^1^Median and 10 and 90th percentiles were calculated using untransformed IGF measures.

^2^For IGFBP-1, N=91 samples were below the lower limit of detection (LLOD) and were reassigned as ½ LLOD (0.025).

^3^P-value for comparison of IGF levels by menopausal status. P-values <0.05 are in bold font.

Table S4. Age- and BMI-adjusted^2^ linear regression estimates for associations between IGF measures (pg/mL) and mammographic density measures, premenopausal women (N= 193)

| IGF measures | % MD-V^1^ | |  | % MD-A | |  | Absolute MD-V | |  | Absolute MD-A | |  | Non-Dense Volume | |  | Non-Dense Area | |
| --- | --- | --- | --- | --- | --- | --- | --- | --- | --- | --- | --- | --- | --- | --- | --- | --- | --- |
|  | β | P^4^ |  | β | p |  | β | p |  | β | p |  | β | p |  | β | p |
| IGF-I | 0.77 | 0.77 |  | 0.30 | 0.93 |  | 3.19 | 0.62 |  | 2.31 | 0.56 |  | -13.39 | 0.24 |  | -3.47 | 0.45 |
| IGFBP-1^3^ | 36.72 | 0.17 |  | 41.24 | 0.22 |  | -33.55 | 0.62 |  | 21.45 | 0.61 |  | -252.78 | **0.03** |  | -77.03 | 0.11 |
| IGFBP-2 | 2.16 | **0.0004** |  | 2.24 | **0.004** |  | -0.80 | 0.60 |  | 1.54 | 0.11 |  | -11.16 | **<.0001** |  | -3.46 | **0.002** |
| IGFBP-3 | -0.26 | 0.07 |  | -0.37 | **0.04** |  | 0.33 | 0.35 |  | -0.15 | 0.51 |  | 1.11 | 0.08 |  | 0.50 | 0.05 |
| IGFBP-4 | -7.17 | **0.02** |  | -5.64 | 0.15 |  | -7.02 | 0.36 |  | -5.37 | 0.26 |  | 25.10 | 0.07 |  | 5.56 | 0.32 |
| IGFBP-5 | -0.08 | 0.91 |  | 0.80 | 0.41 |  | -0.78 | 0.68 |  | 0.61 | 0.61 |  | -2.10 | 0.54 |  | -2.09 | 0.13 |
| IGFBP-6 | 2.56 | 0.30 |  | 3.83 | 0.22 |  | 10.57 | 0.09 |  | 6.16 | 0.11 |  | -7.20 | 0.51 |  | -2.93 | 0.51 |
| IGFBP-7 | 1.38 | 0.72 |  | 0.20 | 0.97 |  | -5.07 | 0.59 |  | -3.41 | 0.56 |  | -10.90 | 0.52 |  | -4.53 | 0.51 |
| IGF-I: IGFBP-1 | 0.00 | 0.71 |  | 0.00 | 0.28 |  | 0.00 | 0.99 |  | 0.00 | 0.30 |  | 0.00002 | 0.65 |  | 0.00001 | 0.56 |
| IGF-I: IGFBP-2 | -0.21 | **0.03** |  | -0.24 | 0.05 |  | 0.01 | 0.97 |  | -0.17 | 0.27 |  | 0.75 | 0.08 |  | 0.24 | 0.16 |
| IGF-I: IGFBP-3 | 5.46 | 0.08 |  | 6.23 | 0.11 |  | 0.69 | 0.93 |  | 5.93 | 0.22 |  | -36.24 | **0.01** |  | -12.50 | **0.02** |
| IGF-I: IGFBP-4 | 0.07 | 0.36 |  | 0.01 | 0.91 |  | 0.13 | 0.48 |  | 0.06 | 0.59 |  | -0.48 | 0.14 |  | -0.08 | 0.53 |
| IGF-I: IGFBP-5 | -0.04 | 0.87 |  | -0.28 | 0.33 |  | 0.36 | 0.52 |  | -0.05 | 0.88 |  | -0.16 | 0.87 |  | 0.39 | 0.34 |
| IGF-I: IGFBP-6 | -0.06 | 0.70 |  | -0.15 | 0.40 |  | -0.20 | 0.58 |  | -0.14 | 0.53 |  | -0.47 | 0.46 |  | -0.07 | 0.78 |
| IGF-I: IGFBP-7 | -0.05 | 0.45 |  | -0.05 | 0.58 |  | 0.06 | 0.70 |  | 0.03 | 0.75 |  | -0.02 | 0.96 |  | 0.02 | 0.87 |
| IGF-I: Total IGFBP | 4.27 | 0.31 |  | 4.12 | 0.43 |  | 4.41 | 0.67 |  | 5.94 | 0.35 |  | -32.39 | 0.08 |  | -9.79 | 0.19 |

IGF, insulin-like growth factor; IGFBP, IGF binding protein; MD-V, Mammographic density-volume; MD-A, MD-area

^1^Mammographic density measures were square-root transformed.

^2^Models were adjusted for categorical Age (Premenopausal: <45, 45-49, ≥50; Postmenopausal: >54, 55-59, ≥60) and BMI (<25, 25-29.9, and >30)

^3^For IGFBP-1, N=91 samples were below the lower limit of detection (LLOD) and were reassigned as ½ LLOD (0.025).

^4^P-value for comparison of IGF levels by menopausal status. P-values <0.05 are in bold font.

Table S5. Age- and BMI-adjusted^2^ linear regression estimates for associations between IGF measures (pg/mL) and mammographic density measures, postmenopausal women (N= 103)

|  | % MD-V^1^ | |  | % MD-A | |  | Absolute MD-V | |  | Absolute MD-A | |  | Non-Dense Volume | |  | Non-Dense Area | |
| --- | --- | --- | --- | --- | --- | --- | --- | --- | --- | --- | --- | --- | --- | --- | --- | --- | --- |
| IGF measures | β | P^4^ |  | β | p |  | β | p |  | β | p |  | β | p |  | β | p |
| IGF-I | 4.06 | 0.31 |  | 6.84 | 0.19 |  | -1.67 | 0.86 |  | 7.66 | 0.24 |  | -35.89 | 0.08 |  | -15.75 | 0.05 |
| IGFBP-1^3^ | 65.64 | 0.08 |  | -29.73 | 0.55 |  | -92.60 | 0.31 |  | -70.11 | 0.26 |  | -474.06 | **0.02** |  | -37.51 | 0.62 |
| IGFBP-2 | 2.33 | **0.002** |  | 0.56 | 0.58 |  | -1.59 | 0.39 |  | -0.49 | 0.70 |  | -14.22 | **0.0003** |  | -2.02 | 0.19 |
| IGFBP-3 | 0.04 | 0.81 |  | 0.32 | 0.10 |  | -0.22 | 0.55 |  | 0.43 | 0.08 |  | -0.93 | 0.24 |  | -0.55 | 0.07 |
| IGFBP-4 | -4.07 | 0.17 |  | -4.62 | 0.24 |  | 5.02 | 0.49 |  | -1.37 | 0.78 |  | 23.74 | 0.13 |  | 12.99 | **0.03** |
| IGFBP-5 | 0.09 | 0.94 |  | 0.56 | 0.74 |  | 0.82 | 0.79 |  | 1.37 | 0.52 |  | -2.20 | 0.74 |  | -1.85 | 0.47 |
| IGFBP-6 | -3.23 | 0.22 |  | -5.63 | 0.11 |  | 3.27 | 0.61 |  | -4.87 | 0.27 |  | 28.43 | **0.04** |  | 13.29 | **0.01** |
| IGFBP-7 | 2.84 | 0.62 |  | -10.66 | 0.16 |  | 15.85 | 0.25 |  | -17.11 | 0.07 |  | 14.29 | 0.64 |  | 16.37 | 0.16 |
| IGF-I: IGFBP-1 | -0.00002 | 0.07 |  | 0.00 | 0.61 |  | <0.001 | 0.98 |  | <0.001 | 0.20 |  | 0.0001 | 0.11 |  | 0.00 | 0.95 |
| IGF-I: IGFBP-2 | -0.13 | 0.18 |  | 0.17 | 0.18 |  | -0.05 | 0.82 |  | 0.29 | 0.07 |  | 0.46 | 0.37 |  | -0.19 | 0.34 |
| IGF-I: IGFBP-3 | 6.58 | 0.20 |  | -0.97 | 0.89 |  | 8.11 | 0.52 |  | -3.97 | 0.64 |  | -35.83 | 0.19 |  | -8.21 | 0.43 |
| IGF-I: IGFBP-4 | 0.19 | 0.14 |  | 0.33 | 0.05 |  | -0.09 | 0.76 |  | 0.32 | 0.14 |  | -1.22 | 0.07 |  | -0.65 | **0.01** |
| IGF-I: IGFBP-5 | 0.41 | 0.24 |  | 0.56 | 0.22 |  | 0.12 | 0.89 |  | 0.56 | 0.33 |  | -2.49 | 0.17 |  | -0.93 | 0.18 |
| IGF-I: IGFBP-6 | 0.21 | 0.17 |  | 0.32 | 0.11 |  | -0.21 | 0.57 |  | 0.30 | 0.24 |  | -1.97 | **0.01** |  | -0.88 | **0.004** |
| IGF-I: IGFBP-7 | 0.04 | 0.70 |  | 0.25 | 0.08 |  | -0.12 | 0.65 |  | 0.37 | **0.04** |  | -0.68 | 0.25 |  | -0.41 | 0.07 |
| IGF-I: Total IGFBP | 6.79 | 0.30 |  | 3.94 | 0.65 |  | 8.52 | 0.60 |  | 2.84 | 0.80 |  | -43.74 | 0.21 |  | -16.74 | 0.21 |

IGF, insulin-like growth factor; IGFBP, IGF binding protein; MD-V, Mammographic density-volume; MD-A, MD-area

^1^Mammographic density measures were square-root transformed.

^2^Models were adjusted for categorical Age (Premenopausal: <45, 45-49, ≥50; Postmenopausal: >54, 55-59, ≥60) and BMI (<25, 25-29.9, and >30)

^3^For IGFBP-1, N=91 samples were below the lower limit of detection (LLOD) and were reassigned as ½ LLOD (0.025).

^4^P-value for comparison of IGF levels by menopausal status. P-values <0.05 are in bold font.

Table S6. Factor loadings for IGF analytes, overall and by menopausal status

|  | Overall | |  | Premenopausal | | |  | Postmenopausal | | |
| --- | --- | --- | --- | --- | --- | --- | --- | --- | --- | --- |
|  | ^1^Factor loadings | |  | *Factor loadings | | |  | *Factor loadings | | |
| Analytes | Factor 1 | Factor 2 |  | | Factor 1 | Factor 2 |  | | Factor 1 | Factor 2 |
| IGFBP-3 | *79 | 5 |  | | *84 | 2 |  | | *74 | -29 |
| IGF-I | *68 | -6 |  | | *71 | 8 |  | | *74 | 1 |
| IGFBP-6 | 22 | 11 |  | | 32 | 10 |  | | 47 | -20 |
| IGFBP-1 | *-61 | 13 |  | | *-56 | 27 |  | | *-57 | -23 |
| IGFBP-2 | *-65 | -1 |  | | *-59 | 14 |  | | *-65 | -29 |
| IGFBP-4 | -4 | *79 |  | | -3 | *80 |  | | -8 | *77 |
| IGFBP-7 | -6 | *79 |  | | 4 | *78 |  | | -4 | *68 |
| IGFBP-5 | 10 | *68 |  | | -5 | *76 |  | | 11 | *58 |
| Variance explained | 53.04% | 46.96% |  | | 53.81% | 46.19% |  | | 55.93% | 44.07% |

IGF, insulin-like growth factor; IGFBP, IGF binding protein;

^1^Factors were created using overall women. When the factors were extracted using the dataset stratified by menopausal status the factors were the same.

*IGF measures that were significantly involved in each factor.

Table S7. Age- and BMI-adjusted^1^ linear regression estimates for the relation of IGF factors (in quintiles) with mammographic density measures

|  | % MD-V^1^ | |  | % MD-A | |  | Absolute MD-V | |  | Absolute MD-A | |  | Non-Dense Volume | |  | Non-Dense Area | |
| --- | --- | --- | --- | --- | --- | --- | --- | --- | --- | --- | --- | --- | --- | --- | --- | --- | --- |
| Premenopausal Women | β | p^3^ |  | β | p |  | β | p |  | β | p |  | β | p |  | β | p |
| Factor 1^2^ | -0.16 | **0.02** |  | -0.18 | **0.04** |  | 0.2 | 0.28 |  | -0.05 | 0.67 |  | 0.24 | 0.05 |  | 0.68 | **0.02** |
| Factor 2 | -0.003 | 0.96 |  | -0.01 | 0.91 |  | -0.12 | 0.49 |  | -0.07 | 0.49 |  | -0.09 | 0.44 |  | -0.11 | 0.69 |
| Postmenopausal Women |  | |  |  | |  |  | |  |  | |  |  | |  |  | |
| Factor 1 | -0.21 | **0.013** |  | 0.02 | 0.87 |  | 0.11 | 0.62 |  | 0.18 | 0.19 |  | 0.09 | 0.56 |  | 1.04 | **0.01** |
| Factor 2 | -0.02 | 0.74 |  | -0.1 | 0.35 |  | 0.18 | 0.40 |  | -0.08 | 0.55 |  | 0.15 | 0.35 |  | 0.14 | 0.74 |

MD-V, Mammographic density-volume; MD-A, MD-area

^1^Models were adjusted for categorical Age (Premenopausal: <45, 45-49, ≥50; Postmenopausal: >54, 55-59, ≥60) and BMI (<25, 25-29.9, and >30)

^2^Factor-1 was positively correlated with IGF-I and IGFBP-3 and inversely correlated with IGFBP-1 and IGFBP-2; Factor-2 was positively correlated with IGFBP-4 IGFBP-5, and IGFBP-7. Quintiles cutpoints for factor 1 and factor 2 were created based on their distributions in premenopausal and postmenopausal women separately.

^3^P-values <0.05 are in bold font.
